# Three-dimensional echocardiography of the tricuspid valve

**DOI:** 10.3389/fcvm.2023.1114715

**Published:** 2023-03-20

**Authors:** Zachary T. Jost, Nishank P. Nooli, Ahmed E. Ali, Vijayadithyan Jaganathan, Navin C. Nanda

**Affiliations:** ^1^ Department of Internal Medicine, University of Alabama at Birmingham, Birmingham, AL, United States; ^2^ Division of Cardiothoracic Anesthesia, Department of Anesthesiology and Perioperative Medicine, University of Alabama at Birmingham, Birmingham, AL, United States; ^3^ Division of Cardiovascular Disease, Department of Medicine, University of Alabama at Birmingham, Birmingham, AL, United States

**Keywords:** echocardiography, three-dimensional echocardiography, tricuspid valve, two-dimensional echocardiography, transcatheter tricuspid valve repair, transcatheter tricuspid valve replacement, three-dimensional printing

## Abstract

Due to the proportionally high mortality rates associated with isolated tricuspid valve surgery, the invasive treatment of such pathology, historically, has been left largely unaddressed. Recently, there has been an appreciation for the mortality and morbidity of tricuspid valve disease, giving rise to the movement towards identifying less invasive, transcatheter approaches for treatment. Due to the technical complexity of these procedures along with the uniqueness and variability of tricuspid valve anatomy, a better appreciation of the tricuspid valve anatomy and pathology is required for pre-procedural planning. While two-dimensional echocardiography serves as the initial non-invasive modality for tricuspid valve evaluation, three-dimensional echocardiography provides a complete *en face* view of the tricuspid valve and surrounding structures, as well contributes further information regarding disease etiology and severity. In this review, we discuss the utility of three-dimensional echocardiography as a supplement to two-dimensional imaging to better assess tricuspid valve disease and anatomy to aide in future innovative therapies.

## Introduction

**Figure 1:**
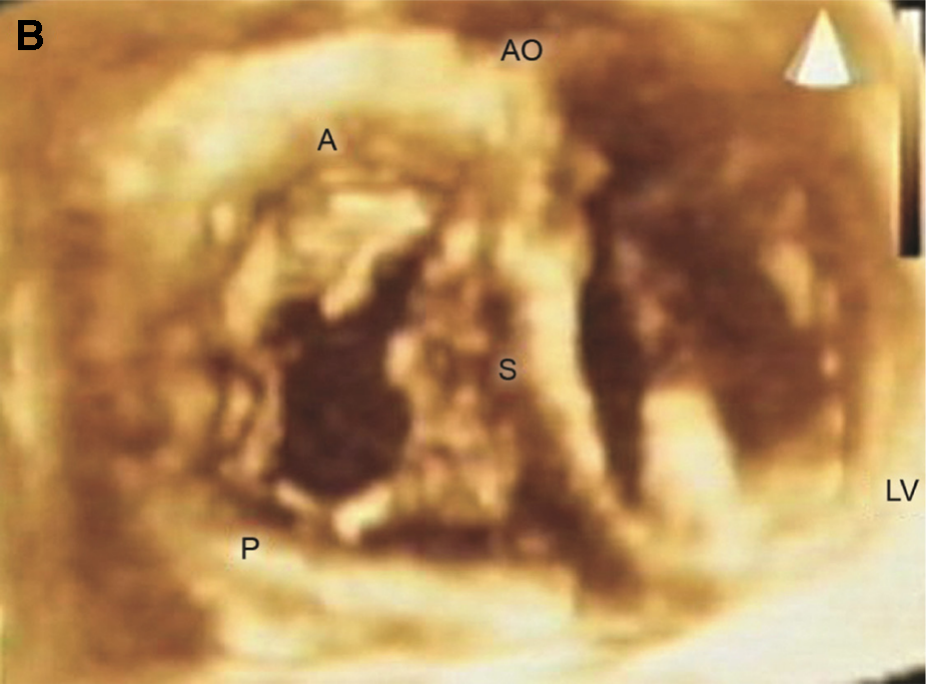
*En face* view of the TV showing all three leaflets in the open positions. A = anterior leaflet; AO= aorta; LV= left ventricle; P = posterior leaflet; S = septal leaflet. Reproduced with permission from Pothineni K et al (23).

**Figure 2:**
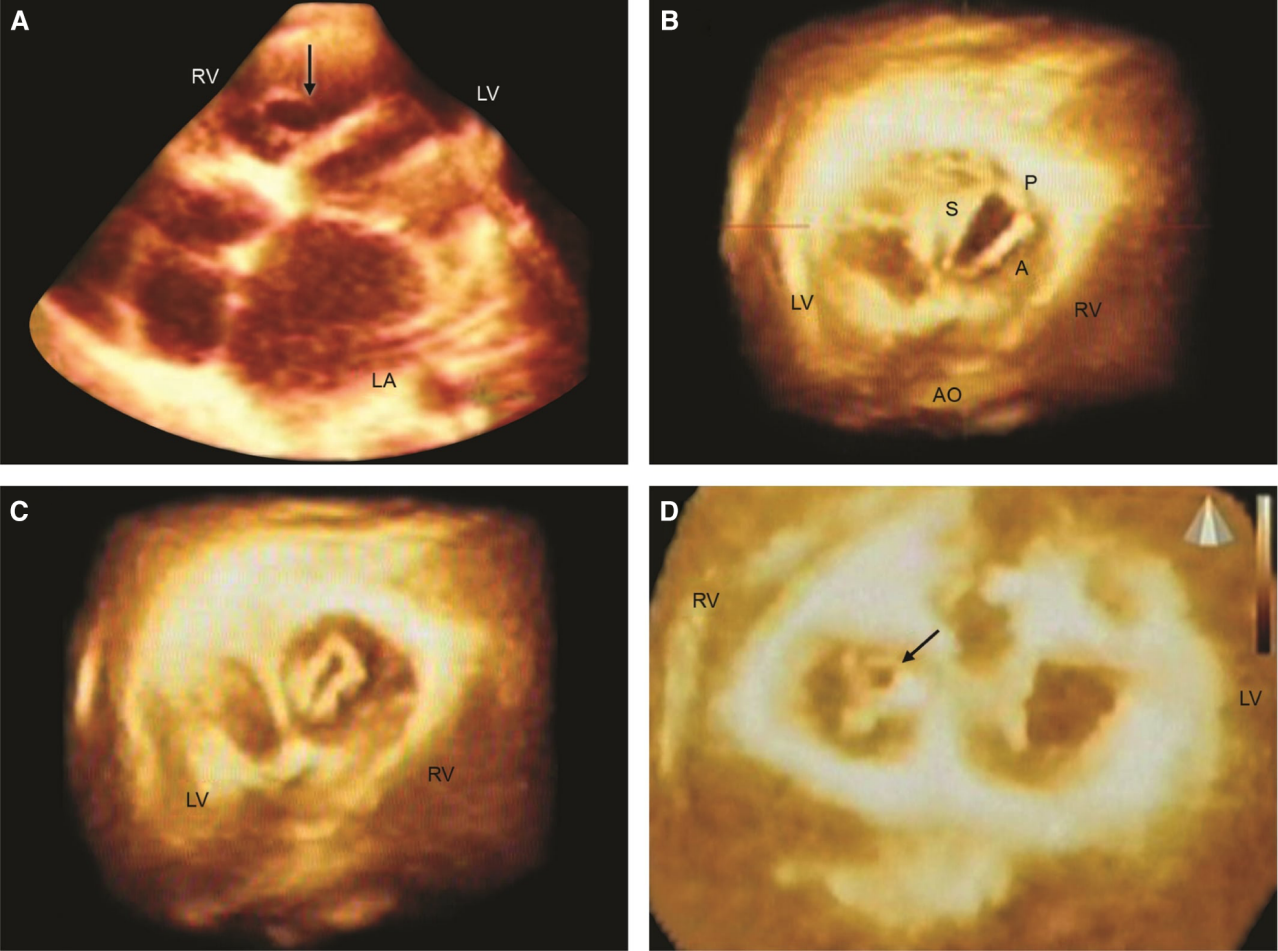
Live/real time three-dimensional transthoracic echocardiography in rheumatic tricuspid valve (TV) stenosis and regurgitation (TR). A: The arrow points to the TV orifice in a patient with TV stenosis. The orifice area measured 2.02 cm^2^ in diastole. B,C: *En face* views in another patient with mild TV stenosis but severe TR. The TV orifice area (B) measured 2.4 cm^2^ in diastole. Systolic frame (C) shows noncoaptation of TV leaflets in the same patient as (B). This measured 0.4 cm^2^ and resulted in severe TR as assessed by two-dimensional color Doppler. D: En face view from the ventricular aspect in a third patient with rheumatic heart disease showing systolic non coaptation (arrow) of the TV. LA = left atrium; RV = right ventricle. Other abbreviations as in previous figures. Reproduced with permission from Pothineni K et al (23).

**Figure 3:**
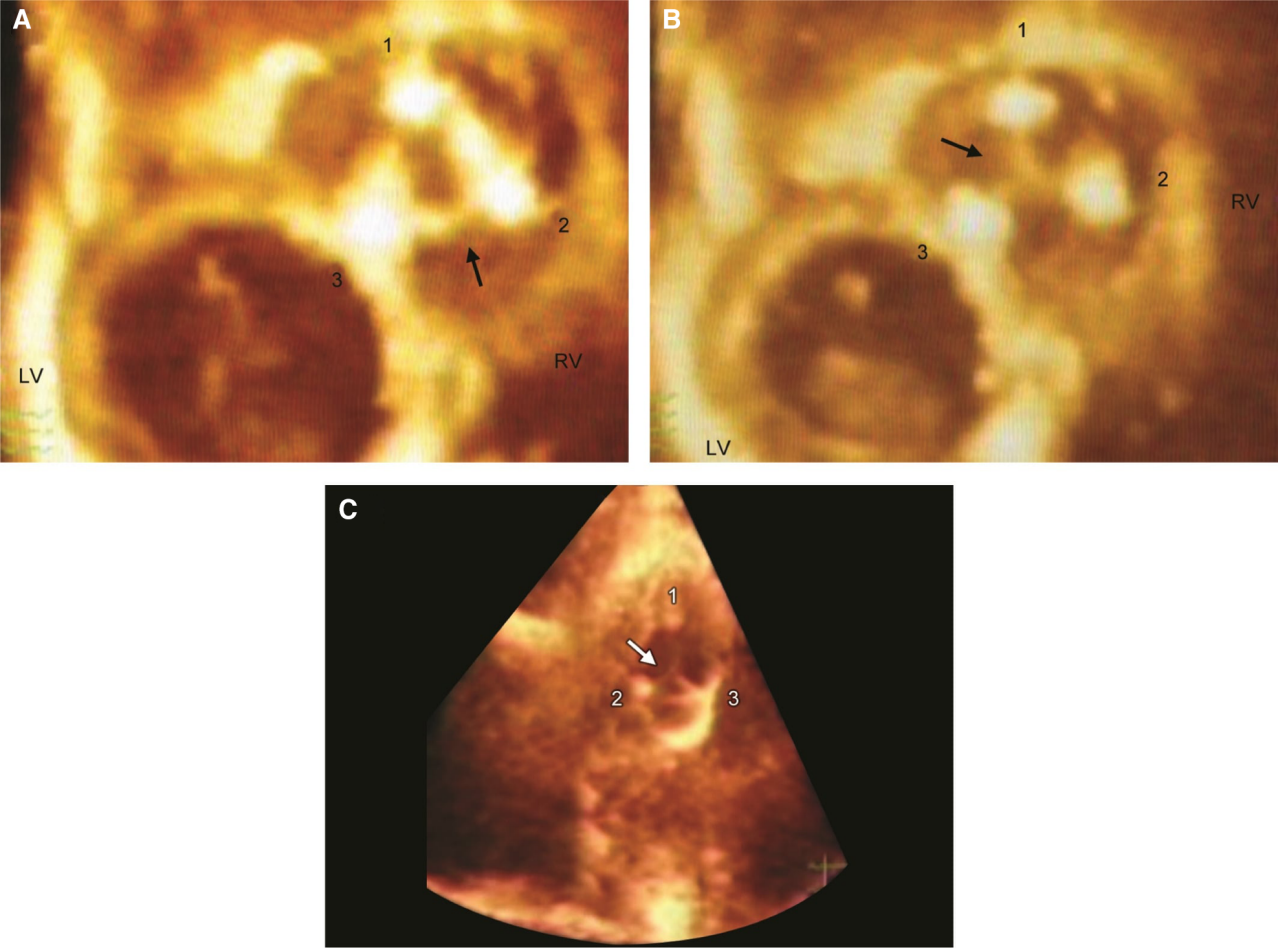
Live/real time three-dimensional transthoracic echocardiography of TV prosthesis. A,B: Normal bioprosthetic TV leaflets (arrow) in open (A) and closed (B) positions. Numbers 1, 2, and 3 represent the three struts of the prosthetic valve. C: Arrow shows systolic noncoaptation of bioprosthetic leaflets in another patient. Abbreviations as in previous figures. Reproduced with permission from Pothineni at al (23).

**Figure 4:**
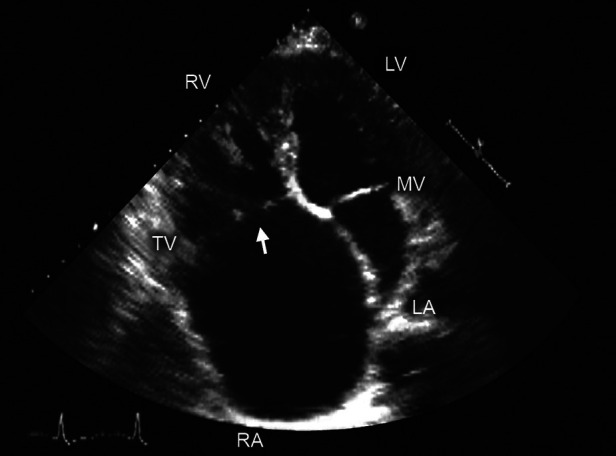
Two-dimensional transthoracic echocardiography. Apical 4 chamber view shows noncoaptation of tricuspid valve (TV) leaflets (arrow) due to a dilated annulus-an important consideration in procedural planning for TV disease. Abbreviations as in previous figures. Reproduced with permission from Murray, et al. (5).

**Figure 5:**
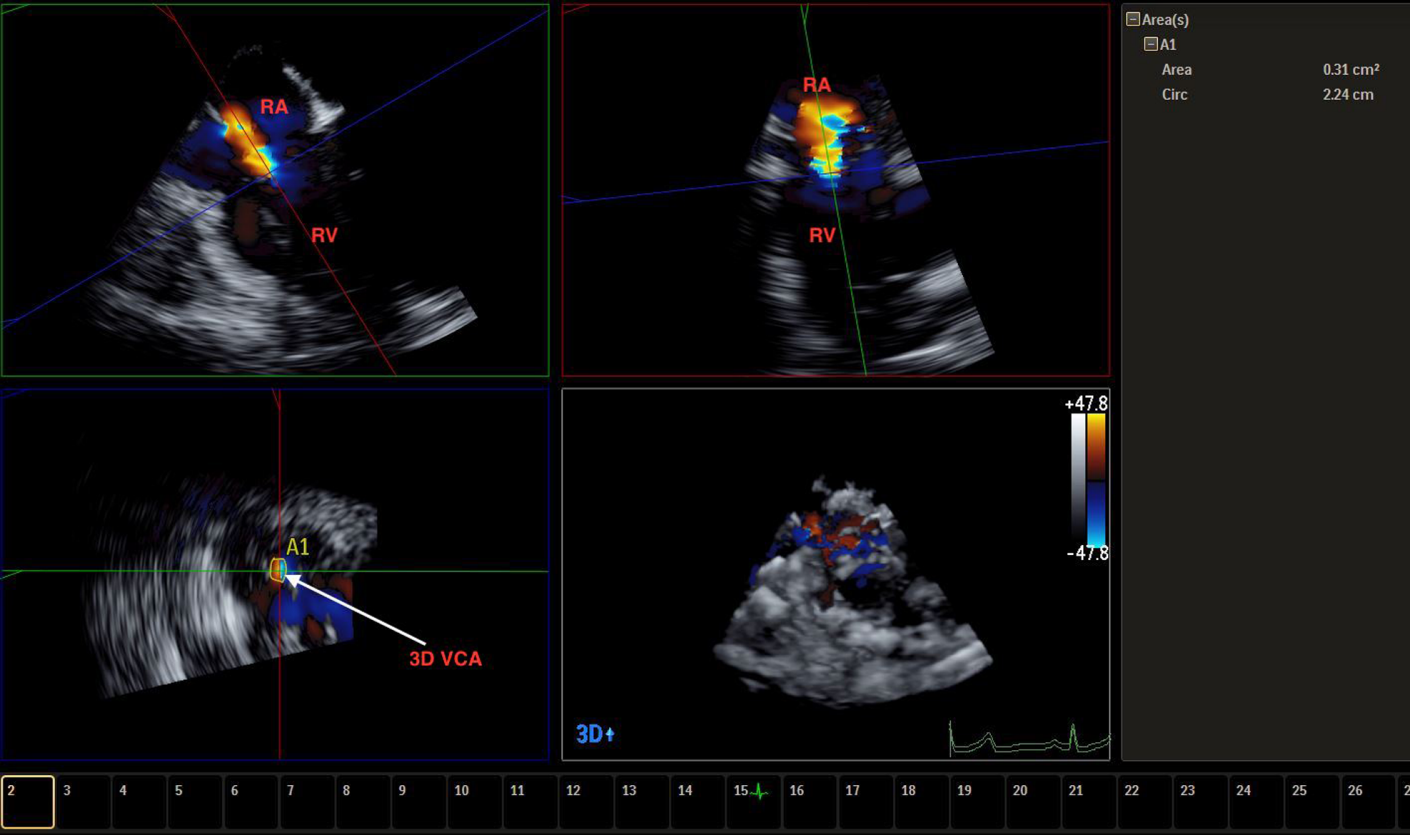
Live/real time transesophageal echocardiographic assessment of TR. The color Doppler data set obtained from a four chamber view was cropped to depict the VC which measured 0.41 cm^2^ in area (A). Abbreviations as in previous figures.

**Figure 6:**
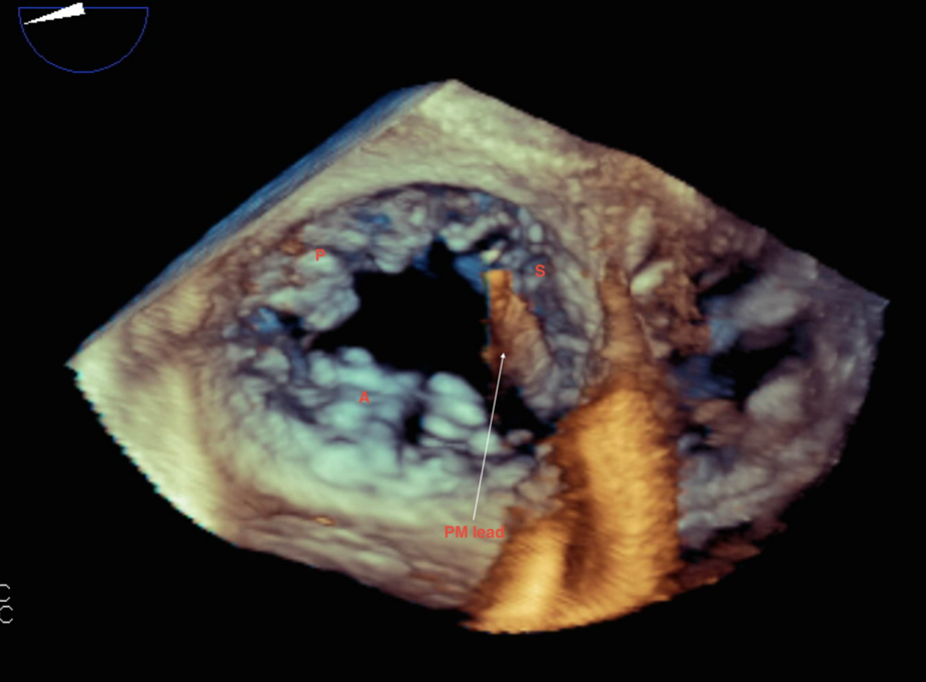
Live/real time transesophageal echocardiography. The arrow points to a pacemaker (PM) lead impinging on the septal leaflet (S) of the tricuspid valve. This caused restricted motion of the septal leaflet resulting in severe TR. Findings were confirmed at surgery. Abbreviations as in previous figures.

Recently, the morbidity and mortality associated with tricuspid valve (TV) pathology has become more appreciated. Five-year survival is < 30% with moderate to severe tricuspid regurgitation (TR), which is associated with increased mortality independent of factors such as ejection fraction and pulmonary artery systolic pressure (1–3). Additionally, worsening of underlying TR after surgery of left sided valves has been documented (4–7). Due to the significant risk that TV disease poses, focus has shifted towards treatment of the TV disease from both surgical, and more recently, transcatheter approaches (5). Surgical TV repair has been linked to a high mortality rate (8), thus prompting the need for minimally invasive options. As many new transcatheter devices and surgical options are being brought to the market, the need for an enhanced knowledge of the TV anatomy and disease severity becomes important for success of these technically complex procedures. In this review, we discuss the role of 3-dimensional (3D) echocardiography as a supplement to 2-dimensional (2D) echocardiography in assessing TV anatomy and pathology (Figures 1–6).

## Tricuspid valve anatomy

The TV consists of three leaflets: the anterior leaflet (AL), which is largest and quadrangular, the posterior leaflet (PL), which is smallest and triangular, and the septal leaflet (SL), which is semicircular in shape with scalloped indentations (9). Hahn, et al. further assessed tricuspid valve leaflets *via* 2D and 3D transesophageal echocardiography (TEE) and determined that the TV has three well delineated leaflets in ∼54% of patients, four leaflets in ∼39% of patients (two posterior leaflets are most frequent), and two leaflets in ∼5% of patients (10). There are multiple, small papillary muscles in the TV apparatus and often, there are accessory chordal attachments to the right ventricle (RV) free wall and moderator band, particularly with the SL (9). Unlike the mitral valve, each TV leaflet is attached to one set of papillary muscles, increasing the risk of wide noncoaptation with annular and RV dilation (9). The TV is oval and nonplanar (with the most ovoid shape occurring at end-diastole) (11) and is 20% larger than the MV (9).

In addition to understanding the anatomy and physiology of the TV in diseased conditions, 3D echocardiography has greatly contributed to our knowledge of normal TV annulus (A) geometry (11, 12). TVA measurements obtained *via* 2D echocardiography have been shown to be consistently underestimated in all views compared to 3D echocardiography (13), owing to the complex geometry of the TVA that 2D imaging is unable to accurately assess. In regards to diseased patients, recent studies using 3D transthoracic echocardiography (TTE) have shown that the TVA evolves to become more planar and circular in patients with secondary TR (STR) (14). The TVA is close in proximity to the right coronary artery, coronary sinus, mitral valve, and aortic valve, all of which are important structures to consider intraprocedurally. Additionally, the TVA is angulated in relation to the superior and inferior vena cava, which can pose technical difficulties during transcatheter procedures (9). The large size of the TV, the significant variability in its shape and dimensions, and the characteristics of the valve itself create difficulty in performing percutaneous repair or replacement (5). In addition to 2D, 3D echocardiography has played a vital role in understanding TV morphology (5, 10, 14–17).

## Etiology of TV disease

The etiology of TV disease can be classified into primary, referring to pathologic changes to the TV leaflets (Table 1), or secondary, referring to pathologic changes to the surrounding heart structures (Table 2) (18). Additionally, STR has been further subdivided into atrial, characterized by insufficient leaflet coverage of a dilated TVA, and ventricular, characterized by insufficient leaflet coaptation in the setting of apical displacement with leaflet tethering (19). Finally, cardiac implantable electronic device(CIED) lead-induced TR has been suggested as a third phenotype due to the nuance on whether to classify this as primary or secondary (19).

**Table 1:** Causes of primary TV disease.

Rheumatic valve disease: Commissural fusion, thick TV leaflets with restricted mobility, thickened/shortened chordae, TV stenosis and/or regurgitation
Infective/marantic endocarditis, masses, thrombi, fibroelastoma on the TV
Myxomatous degeneration/TV prolapse
TV or RV papillary muscle injury or chordae rupture (chest wall trauma, pacemaker or ICD lead induced, endomyocardial biopsy, RV infarction)
Congenital (Ebstein's anomaly, TV atresia, TV clefts, TV dysplasia, double orifice TV, unguarded TV orifice)
Carcinoid syndrome: Thickened/restricted TV leaflets. Carcinoid deposits on chamber/IVC wall
Loeffler syndrome: Thickened TV

ICD, implantable cardioverter defibrillator; IVC, inferior vena cava; RV, right ventricle. Reproduced with permission from Murray, et al with modifications (5).

**Table 2:** Causes of secondary TV disease.

Primary and/or secondary pulmonary hypertension causing RV/RA/TVA dilatation
RV cardiomyopathy: dilated, ischemic, and arrhythmogenic RV dysplasia
Pulmonary valve/artery stenosis
Left sided disease: LV dysfunction or mitral/aortic valvular disease resulting in pulmonary hypertension
Congenital: Left-to-right shunt (ASD, VSD, anomalous pulmonary venous return), post-Tetralogy of Fallot repair with severe pulmonary regurgitation
Atrial fibrillation resulting in LA/RA dilation

LA, left atrium; LV, left ventricle; RA, right atrium; RV, right ventricle; TVA, tricuspid valve annulus; ASD, Atrial septal defect; VSD, Ventricular septal defect. Reproduced with permission from Murray, et al with modifications (5).

## Procedural considerations for TV disease

Due to the high mortality associated with TV disease and with traditional TV surgery, innovative therapies to repair or replace the TV have been recently brought to market (5). There currently are multiple, albeit some experimental, options to perform transcatheter TV repair(TTVr) (5). Transcatheter approach for tricuspid valve repair has been shown to be beneficial in some patients with STR, but is not appropriate in patients with a TV coaptation gap > 6–8 mm, in patients with primary causes of TV disease, or in patients with tricuspid stenosis (TS), in which a surgical approach has traditionally be recommended (1). Recently, Goldberg et al. have suggested a transcatheter tricuspid valve replacement (TTVR) approach, which may address these deficiencies with TTVr (1). The two categories of TTVR include orthotopic, where the valve is implanted at the TVA, and heterotopic, in which the valve is deployed in one or both of the vena cavae in order to ameliorate the symptoms of right heart failure (1). Potential complications of TTVR include device malfunction, paravalvular leak, valve embolism or thrombus, and acute RV failure due to sudden onset RV pressure increase (1). Additionally, multiplanar reconstruction using 3D echocardiography can help guide deployment of TV edge-to-edge repair (20). These important considerations underscore the importance of accurate assessment of TV and RV anatomy and pathology prior to implementation of these approaches. As guidelines suggest a prompter TV intervention in patients with progressive RV dysfunction (21), accurately assessing the size and function of the RV with 3D imaging has been shown to be of importance (22). Post-procedurally, 3D imaging has been shown to be effective in evaluating prosthetic valves and paravalvular leaks, as it provides an *en face* view of the anatomy and function of all three TV prosthetic leaflets, which 2D imaging is mostly unable to accomplish (23).

## Tricuspid valve echocardiography

Imaging of the TV should be performed by 2DTTE/3DTTE and 2DTEE/3DTEE, if indicated, to evaluate the valve (21, 24, 25), chamber dimensions (26), and RV ejection fraction (22). Due to the uniqueness and variability of the TV (Table 3), 2DTTE is able to visualize all three leaflets simultaneously in only 5%–10% of patients; while simultaneous visualization with 3DTTE is achievable in 85%–90% of patients (27). Additionally, the onset of 3D printing has been conceptualized to better facilitate procedural planning for tricuspid valve procedures (5).

**Table 3:** Challenges associated with 2D assessment of the TV.

TV is largest in comparison to other valves
TV may have between 2 and 4 leaflets and varies amongst patients
There is significant variation amongst the shape and size of the TV
Nonplanar geometry of the TVA
2D planes often fail to visualize all three leaflets, requiring multiple views to be obtained, in addition to increased risk of misidentifying leaflets
There is the need to mentally visualize a 3D picture from 2D planes, increasing interobserver variability

Abbreviations as in Table 1.

Assessment of the TV involves a comprehensive imaging approach using 2D TTE/TEE, 3D TTE/TEE, color flow Doppler, and in some instances, cardiac magnetic resonance imaging (25). Most of these measures are qualitative or semiquantitative in nature (25). While 2DTTE remains to have a major impact on valvular assessment, the role of 3D assessment has become increasingly recognized (21, 24, 25, 28, 29). In addition to the limitations associated with 2DTTE, 3DTTE allows for evaluation of entire leaflets, including the subvalvular structures, other surrounding structures, and right heart chambers (5, 22). 3D imaging of the TVA has also been compared to cardiac computed tomography (CCT) imaging and shown to underestimate TVA parameters, but these differences were clinically negligible (11, 12), without having to undergo the extensive imaging protocols of CCT as well as reducing the risk of radiation and contrast exposure. Although technical skills are required to obtain a 3D image, many modern 2D transducers are able to acquire 3D images by using 2D planes. *En face* views of the TV from both the atrial and ventricular short axis can be obtained *via* 3D imaging, which is usually not possible with 2D imaging (5). *En face* views allow for better assessment of TV anatomy, anatomic relationships, and leaflet function (5). Additionally, subvalvular structures such as chordae tendinae, papillary muscles, and moderator band can be seen more clearly with 3D TTE (23). 3D TEE and 3D TTE have their distinct advantages. While 3D TEE avoids the poor acoustic windows that TTE may encounter and provides higher resolution, it can be susceptible to dropout artifacts and experience difficulty visualizing anterior structures that are adjacent to the probe such as the TV.

3DTTE offers further information in regard to the severity and etiology of TR. 3DTTE has been shown to identify structural deficits as the cause of TR (30, 31). For example, 3DTTE has been shown to be effective in evaluating the size and function of the right atrium and right ventricle and their association with TR etiology and severity (32, 33). Differentiating the etiology of STR has clinical relevance, particularly as the two have different natural histories, with atrial STR exhibiting a more favorable clinical course and response to TTVR (34, 35) Additionally, *en face* visualization of TV leaflets provides further clarity into the size, shape, and location of leaflet defects, including causes congenital in nature, identifying vegetations in endocarditis, localizing prolapse of individual TV leaflets, assessing sites of TV chordae rupture, characterizing TV tumors, and evaluating valvular and atrial thrombi (23, 36–39). Studying the degree of TR associated with CIED implantation has revealed mixed results, and this has been attributed to the limited role of 2D TTE in evaluating these patients, where device leads are fully visualized traversing the TVA in only 15% of patients (40, 41). For patients with CIED who have limited 2DTTE assessment, *en face* visualization with 3D TTE can further characterize the cause and degree of TR. In patients with STR, 3DTTE may assess the size of the non-coaptable TV leaflet area, the extent of tethering, and the degree of displacement of TV leaflets into the RV (5).

3DTTE also offers quantitative assessment of TR, which has been historically difficult to achieve with 2D imaging. One described technique to quantitatively assess TR severity has been to calculate the TV 3D vena contracta (VC) area. Obtaining the velocity time integral of the TR jet *via* continuous wave Doppler and multiplying it by the VC size will provide the volume of regurgitation (5). A 2D VC width ≥ 0.7 cm has been indicative of severe TR, but this only has moderate sensitivity and moderate specificity, as 2D assessment of VC assumes that the TV orifice is circular or elliptical, which has been shown to be inaccurate (42). Rather, 3D VCA ≥ 0.75 cm^2^ has been indicative of severe TR with a high sensitivity and high specificity (5, 42). In comparing VC assessment between 2D and 3D imaging, maximal VC diameter is often larger by 3D imaging (25, 43), and 2D imaging of VCA correlates poorly with the corresponding 3D image (42). 3D VCA correlates well with effective regurgitant orifice area (EROA), moderately well with VC diameter, and weakly with jet area/right atrial area ratio (25, 44). The effective orifice area of the TV is often derived from the 2D evaluation of proximal isovelocity surface area (PISA), but this often requires making an often incorrect assumption of the shape of the flow acceleration, which can often underestimate STR severity (45). A corrected 2D PISA method, using a correction factor obtained from referencing 3D volumetric assessment, has been proposed and shown a more accurate assessment of STR severity (45). Recently, there have been suggestions that the TR grading system span beyond that of severe to include “massive” (3D VCA 95–114 mm^2^) and “torrential” (3D VCA ≥ 115 mm^2^), citing that many patients have evidence of regurgitant jets that progress multiple grades beyond the current definition of severe, although these numerical cutoffs have not been validated (46, 47). This trend would necessitate a more comprehensive approach to identifying TR severity, in which 3DTTE and 3D TEE could play a vital role.

To capture multiple 3D structures in a dataset, volume angle often must be enlarged, except when assessing the TR VC. This causes a reduction in the frame rate, and therefore temporal resolution. Due to this phenomenon, 3D datasets should be acquired from multiple transthoracic transducer positions that best visualize the TV, with focused 3D examinations being used as a complement to 2D imaging (5, 27). An additional limitation of 3DTTE is it often overestimates leaflet thickness, which is due to blurring or amplification of artifacts (27, 48).

To obtain 3D imaging of the TV, one must begin the exam with the most optimized 2D examination of the TV, often requiring multiple acquisitions from both the left and right parasternal, apical, subcostal transducer approaches. Once visualized, the image is switched to live/real time 3D to ensure the TV is in view. Once confirmed, full-volume mode is applied, which allows focus on the TV without surrounding structures, thus acquiring an image with an increased frame rate resulting in enhanced resolution. When viewing the TV *en face*, the septal TV leaflet should be located at the 6 ‘clock position, although recent studies have suggested alternative approaches more anatomically or surgically appropriate (27, 49). Once relevant RV structures are inside the cropping plane, gain, compression, and magnification parameters can be adjusted for optimization of the 3D image. Cropping using the multi-slice view allows for more accurate measurements, since each plane is a 2D representation of a 3D image (23). The atrial perspective in a 3D dataset is often used to evaluate primary TR (degenerative, traumatic, etc.), while the ventricular view is often used to evaluate commissures and the regurgitant and/or stenotic orifice in patients with STR (27). In patients with poor transthoracic windows or in patients undergoing percutaneous and surgical procedures of the TV, both 2DTEE and 3DTEE can be performed.

Despite the benefits of 3D imaging, projecting a 3D image onto a 2D screen can limit accurate assessment of spatial relationships and is less accurate in representing specific tissue characteristics such as tissue calcifications and vegetations (27). 3D printing obtained from 3D imaging has been shown to be feasible, accurate, and useful in gathering further understanding of TV anatomy and physiology (50). The onset of 3D printing has allowed proceduralists to view these images as real time 3D objects, which can assist in preprocedural planning (51, 52). This can be particularly helpful in patients with challenging anatomy or for complex, innovative procedures, but is limited by the quality of the 3D image itself.

## Conclusion

Morbidity and mortality associated with TV disease is no longer lost on clinicians. Due to the complex anatomy of the TV and nonplanar geometry of the TVA, assessing severity of TV disease can be difficult with 2D imaging alone. 3DTTE/TEE as a supplement to 2D imaging has been shown to provide quantitative data regarding TV pathology, as well as enhance innovative procedural interventions of the TV. This literature review has further gathered information on the technique and benefits associated with 3D echocardiography.
